# Strength Degradation and Dynamic Reliability of Damaged Wires in Mine Hoisting Ropes Under Time-Varying Tension

**DOI:** 10.3390/ma19204343

**Published:** 2026-10-15

**Authors:** Kun Huang, Xiangdong Chang, Yuxing Peng, Wei Tang, Zhou Zhou, Hao Lu, Xiuheng Zhang, Gushuo Jiang, Ran Deng

**Affiliations:** 1 School of Mechanical and Electrical Engineering, China University of Mining and Technology, Xuzhou 221116, China; 2 State Key Laboratory of Intelligent Mining Equipment Technology, Xuzhou 221008, China; 3 Research Institute of Surface and Interface Science and Technology for Energy Equipment, Xuzhou 221116, China; 4 Jiangsu Special Equipment Safety Supervision and Inspection Institute (Xuzhou Branch), Xuzhou 221007, China

**Keywords:** wire rope, dynamic response, strength degradation, reliability

## Abstract

Mine hoisting ropes are subjected to time-varying tension, inertial loads, and constraints imposed by their helical structure during service. These factors readily induce normal compression and slight relative sliding between internal wires, thereby causing surface damage, residual strength degradation, and reduced reliability. In this paper, the internal wires of an 18 × 7 + IWS (Independent Wire Strand Core) mine hoisting rope are taken as the research object. A dynamic model of the hoisting rope and an analytical method for the contact load between internal wires are established. Tensile fracture tests are carried out to develop a residual strength degradation model for damaged wires, and a stress-strength interference reliability model is constructed to analyze the dynamic reliability evolution of damaged wires under different transverse loads. The results show that the rope tension fluctuates significantly with the operating stages during hoisting, and the internal wire forces exhibit marked positional differences. The core strand and interlayer contact regions are identified as potential damage-sensitive zones. As the transverse load increases from 80 N to 320 N, the breaking force of damaged wires decreases approximately linearly with increasing fretting cycles, and the strength degradation slope increases from −9.8 to −21.5, indicating that higher transverse contact loads significantly accelerate wire strength degradation. The working stress of the in-service wires approximately follows a normal distribution, with a mean value of 241.63 MPa and a standard deviation of 26.61 MPa. Under transverse loads of 80, 160, 240, and 320 N, the critical cycles at which the wire reliability begins to decrease rapidly are approximately 1.65 × 10^6^, 1.57 × 10^6^, 1.14 × 10^6^, and 7.38 × 10^5^, respectively.

## 1. Introduction

**Figure 1:**
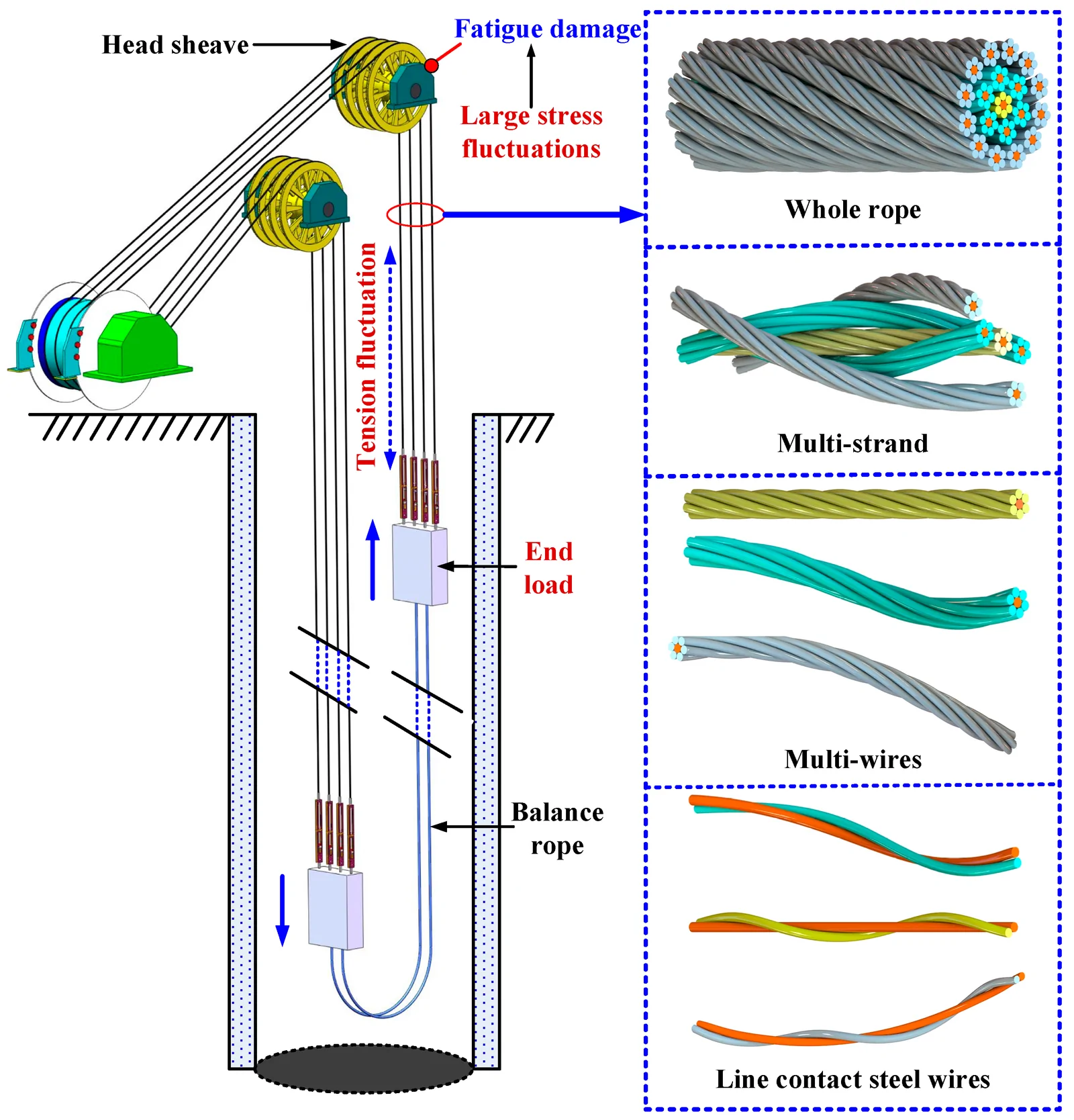
Schematic diagram of mine hoisting and wire rope structure.

In mine hoisting systems, the hoisting rope is one of the key components responsible for load transmission. Its excellent strength, flexibility, and impact-bearing capacity make its health status closely associated with the operational safety of hoisting equipment and the stable production of mines. A wire rope is composed of multiple wires and multilayer strands helically laid with specific lay lengths, lay angles, and lay directions, resulting in complex spatial contact relationships and load transmission paths among internal wires [1,2]. During actual hoisting operation, the wire rope is subjected for long periods to hoisting load, self-weight, inertial force, bending load, and constraints imposed by its internal helical structure, and thus operates under a coupled state of tension, bending, contact compression, and slight relative sliding, as shown in Figure 1. As the conveyance passes through the acceleration, constant speed, and deceleration stages, the rope length, velocity, acceleration, and equivalent stiffness vary continuously, causing the rope tension to exhibit pronounced time-varying fluctuation characteristics [3,4,5,6]. After being transmitted through the helical structure of the wire rope, such dynamic loads induce variations in the tensile force, contact load, and the relative sliding state of internal wires. Under long-term cyclic action, these variations can readily lead to local wire damage, residual strength degradation, and fracture failure [7,8,9,10]. Therefore, revealing the strength degradation behavior of internally damaged wires under hoisting dynamic loads and establishing a reliability evaluation method oriented toward the service process are important issues for the life prediction and safety maintenance of mine hoisting ropes.

Wire rope is not subjected to a constant axial tensile force during hoisting, but exhibits pronounced dynamic response characteristics. Guo et al. [11] investigated the longitudinal dynamic characteristics of wire ropes in a friction hoisting system and their coupling with friction transmission, and pointed out that the tension of the hoisting rope varies dynamically during operation. Wang et al. [12,13] analyzed the dynamic contact and friction transmission behavior between the wire rope and friction lining during deep-mine hoisting, and found that variations in hoisting parameters affect rope tension, contact state, and transmission stability. From the perspectives of dynamic modeling of multi-rope friction hoisting and transverse rope vibration, Ma et al. [14] and Wu et al. [15] demonstrated that hoisting velocity, acceleration, rope length variation, and boundary excitation all influence the dynamic response of wire ropes. Due to the multilayer helical structure of wire ropes, the overall tension is non-uniformly distributed among the core strand, inner strands, and outer strands. Utting et al. [16,17] studied the response of wire rope strands under axial tensile loading and indicated that wire lay angle, contact deformation, and friction affect the stress state of the strands. Ghoreishi et al. [18] analyzed the mechanical response of axially loaded wire rope strands using a three-dimensional finite element model, elucidating the influence of inter-wire contact and geometric nonlinearity on load transmission. Xiang et al. [19] and Yu et al. [20] investigated the interfacial force characteristics of seven-wire strands under axial and transverse loads, and pointed out that transverse loads significantly alter the contact state between wires. Chen et al. [21,22] further established full-contact analytical models for wire rope strands and analyzed the inter-wire contact force and sliding behavior under variable loading conditions.

From the perspectives of failure, strength degradation, and service reliability, wire rope failure is generally the result of the progressive accumulation of local damage in internal wires. Chaplin [23] summarized the typical failure mechanisms of wire ropes and pointed out that fatigue, wear, corrosion, and wire breakage are major causes of performance degradation. Peterka et al. [24] conducted a failure analysis of hoisting ropes and found that dynamic loads, cyclic stresses, wear, and corrosive environments accelerate fatigue damage in wire ropes. Piskoty et al. [25] analyzed the fracture load in a cableway wire rope accident and indicated that transverse pressure and local contact damage can significantly reduce the fracture load-bearing capacity of wire ropes. Reliability evaluation is an important method for predicting the service life of damaged wires and establishing safety criteria. The stress-strength interference theory treats both service stress and material strength as random variables, and can be used to calculate the failure probability and reliability of components [26,27]. Xue et al. [28] introduced stochastic strength degradation into the stress-strength interference reliability model and pointed out that, when material strength degrades with time, traditional static reliability models cannot accurately describe the evolution of failure probability. Li et al. [29] incorporated a time-dependent degradation process and probabilistic limit state into the fatigue life assessment of bridge cable wires, showing that reliability methods considering degradation processes can more reasonably characterize the service life of wire-like components. Ye et al. [30,31] further developed dynamic stress-strength interference models by incorporating impact loads, randomly time-varying strength, and failure criteria into reliability analysis, thereby providing a new approach for reliability calculation of degraded components under complex loading conditions. Gao [32] investigated time-dependent reliability considering clearance, local stiffness, and strength degradation from the perspective of mechanical systems, and pointed out that component reliability decreases nonlinearly during service under the combined effects of random loads and strength degradation.

Previous studies have established important foundations for understanding hoisting dynamics, inter-wire contact, fretting damage, and degradation-based reliability assessment. Building on these studies, the present work integrates calculated time-varying internal-wire loading, experimentally fitted residual tensile-strength degradation, and stress-strength interference analysis within a wire-level assessment framework. The aim is to quantify the effect of transverse contact load on residual wire strength and evaluate how the resulting strength degradation influences wire reliability under time-varying hoisting tension. For an 18 × 7 + IWS mine hoisting rope, dynamic modeling and inter-wire contact analysis are first used to characterize internal-wire loading and guide the selection of fretting test conditions. Tensile tests on the damaged wires are then used to fit residual strength degradation relationships under the selected transverse contact loads. These relationships are subsequently combined with the calculated service stress distribution to evaluate reliability evolution with increasing fretting cycles. The specific contribution is the integration of these modeling and experimental steps to relate hoisting-induced internal-wire loading to measured residual strength degradation and its implications for wire reliability. The resulting framework provides a basis for assessing the remaining load-bearing capacity and service reliability of damaged wires under the investigated conditions.

## 2. Experimental Details

### 2.1. Dynamic Conditions and Geometrical Parameters for Hoisting Rope

**Figure 2:**
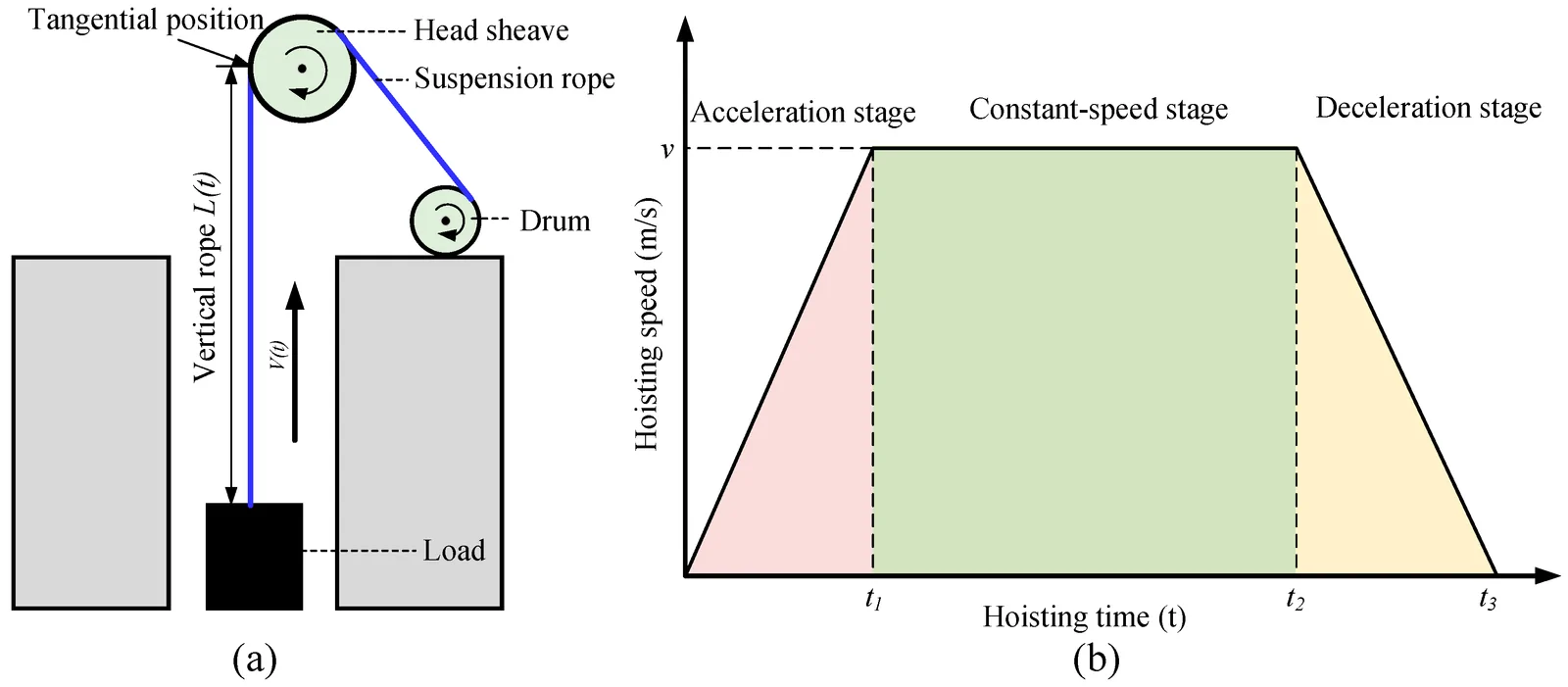
Mine hoisting system and hoisting parameters: (**a**) schematic diagram of the hoisting system, (**b**) hoisting speed variation.

During mine hoisting operations, the hoisting rope is driven by the drum and passes over the head sheave to perform lifting and lowering operations (Figure 2). The operating cycle of a mine hoisting system is typically characterized by three stages, namely acceleration, constant-speed, and deceleration. The Rayleigh method is adopted to transform the weight of the suspended rope and add it to the hoisting load end, with the equivalent value taken as 1/3 of the suspended rope mass. The rope tension *F_R_* at the tangent point on the left side of the head sheave can be calculated using the following equations, where Equation (1), Equation (2), and Equation (3) correspond to the acceleration stage, constant-speed stage, and deceleration stage, respectively [33]:

(1) $${\ddot{F}}_{R}=\left\{E_{R}A_{R}\left(g+a_{1}\right)-\left[\frac{E_{R}A_{R}}{m_{R}+\rho L_{1}\left(t\right)/3}+a_{1}\right]-2a_{1}tF_{R}\right\}/L_{1}\left(t\right)$$

(2) $${\ddot{F}}_{R}=\left\{E_{R}A_{R}g-\frac{E_{R}A_{R}}{m_{R}+\rho L_{2}\left(t\right)/3}-2v_{m}F_{R}\right\}/L_{2}\left(t\right)$$

(3) $${\ddot{F}}_{R}=\left\{E_{R}A_{R}\left(g-a_{2}\right)-\left[\frac{E_{R}A_{R}}{m_{R}+\rho L_{3}\left(t\right)/3}-a_{2}\right]-2\left[v_{m}-a_{2}(t-t_{2})\right]F_{R}\right\}/L_{3}\left(t\right)$$

where *F_R_* is the wire rope tension, ${\ddot{F}}_{R}$ is the second-order derivative of the wire rope tension, *E_R_* is the Young’s modulus of the wire rope, *A_R_* is the total cross-sectional area of the wires in the wire rope, *g* is the gravitational acceleration, *a_1_* and *a_2_* are the hoisting acceleration and deceleration, respectively, *m_R_* is the mass of the hoisting load, *ρ* is the mass per unit length of the wire rope, *L*_1_(*t*), *L*_2_(*t*), *L*_3_(*t*) are the suspended rope lengths between the hoisting load and the head sheave during the acceleration, constant speed, and deceleration stages, respectively, *t* is the hoisting time, *t*_1_ is the end time of the acceleration stage, *t*_2_ is the end time of the deceleration stage, *v_m_* is the maximum hoisting velocity of the wire rope.

The 18 × 7 + IWS wire rope used in this study has a diameter of 15.3 mm, a hoisting depth of 600 m, a terminal load ranging from 1000 kg to 3000 kg, a maximum hoisting velocity of 10.5 m/s, and both acceleration and deceleration of 0.75 m/s^2^. The equivalent elastic modulus of the wire rope is 1 × 10^5^ MPa, the elastic modulus of the steel wires is 2 × 10^5^ MPa, the cross-sectional area of the wire rope is 107.54 mm^2^, and the mass per 100 m is 101 kg. During the hoisting process, 0–15 s corresponds to the acceleration stage, 15–55 s to the constant-speed stage, and 55–70 s to the deceleration stage. To simplify the calculation, the dynamic elastic modulus of the wire rope is approximately taken as its static elastic modulus. Based on the tension differential equations of the hoisting wire rope, the variation in rope tension throughout the entire hoisting process is obtained.

### 2.2. Wire Rope Structural Parameters and Inter-Wire Contact Analysis

An 18 × 7 + IWS wire rope with a diameter of 15.3 mm was selected as the research object. The strand diameter is 3.1 mm, with a core wire diameter of 1.1 mm and an outer wire diameter of 1.0 mm. The wire rope is manufactured in a regular lay configuration. The detailed lay parameters and structural parameters of the wire rope are listed in Table 1.

According to Refs. [1,34], the tensile force of the wire in the *k*-th layer of the *l*-th strand can be calculated as Equation (4):

(4) $$F_{kl}=\frac{{cos}_{\beta_{l}}^{2}\cdot {cos}_{\alpha_{kl}}^{2}\cdot E_{kl}\cdot A_{kl}\cdot S}{\sum_{j=0}^{n}\left[\left(z_{j}\cdot {cos}_{\beta_{j}}^{3}\right)\cdot \left(\sum_{i=0}^{n_{wj}}z_{ij}\cdot {cos}_{\alpha_{kl}}^{3}\right){\cdot E}_{ij}\cdot A_{ij}\right]}$$

where *F_kl_* is the tensile force of the wire in the *k*-th layer of the *l*-th strand, *β_l_* is the lay angle of the *l*-th strand, *α_kl_* is the lay angle of the wire in the *k*-th layer of the *l*-th strand, *E_kl_* is the elastic modulus of the wire in the *k*-th layer of the *l*-th strand, *A_kl_* is the cross-sectional area of the wire in the *k*-th layer of the *l*-th strand, *S* is the strand tensile force, *n* is the number of strand layers, *n_wj_* is the number of wire layers in the strands of the *j*-th layer, *z_j_* is the number of strands in the *j*-th layer, *z_ij_* is the number of wires in the *i*-th layer of the *j*-th strand, *E_ij_* is the elastic modulus of the wires in the *i*-th layer of the *j*-th strand, and *A_ij_* is the cross-sectional area of the wires in the *i*-th layer of the *j*-th strand.

Owing to the different tensile forces acting on the internal wires of the mine hoisting wire rope, different deformation amounts are produced. The relative sliding displacement between the internal wires of the wire rope can be expressed by Equation (5) [2]:

(5) $$∆D_{ij}=\frac{L_{r}\cdot ∆F_{ij}}{cos\beta_{j}\cdot cos\alpha_{ij}\cdot E_{ij}\cdot A_{ij}}$$

where ∆*D_ij_* is the relative sliding displacement generated by the wire in the *i*-th layer of the *j*-th strand, and ∆*F_ij_* is the dynamic load amplitude of the wire in the *i*-th layer of the *j*-th strand.

To obtain the normal load used in the friction and wear tests, the contact load between the wires in the inner and outer strands can be expressed as follows [2]:

(6) $$P_{12}=\frac{F_{j=2}\cdot \frac{cos\beta_{2}\cdot {sin}_{\beta_{2}}^{2}}{r_{2}}\cdot \sqrt{\frac{l_{s2}^{2}+{\left[2\pi \cdot \left(R_{0}+2R_{1}+R_{2}\right)\right]}^{2}}{l_{s2}^{2}+{\left[2\pi \cdot \left(R_{0}+2R_{1}\right)\right]}^{2}}}{\cdot l}_{s2w}}{n_{s2w}}$$

where *P*_12_ is the contact force between the wires of the inner and outer strands, *F_j=_*_2_ is the tensile force of a single outer strand, *β*_2_ is the lay angle of the outer strand, *r*_2_ is the lay radius of the outer strand, *L_s_*_2_ is the lay length of the outer strand, *R*_0_ is the radius of the core strand, *R*_1_ is the radius of the inner strand, *R*_2_ is the radius of the outer strand, *l_s_*_2*w*_ is the lay length of the side wires in the outer strand, *n_s_*_2*w*_ is the number of side wires in the outer strand.

### 2.3. Wire Properties

High-strength cold-drawn steel wires commonly used in mine hoisting wire ropes were selected as the test materials. The steel wires used were made of high-carbon steel, with a chemical composition of 98.53 wt.% Fe, 0.70 wt.% C, 0.55 wt.% Mn, 0.01 wt.% Ni, 0.19 wt.% Si, 0.012 wt.% P, and 0.008 wt.% S. Among its main physical properties, the tensile strength is 2060 MPa, the elastic modulus is 209 GPa, and the yield strength is 1570 MPa.

### 2.4. Tensile Testing Methods

**Figure 3:**
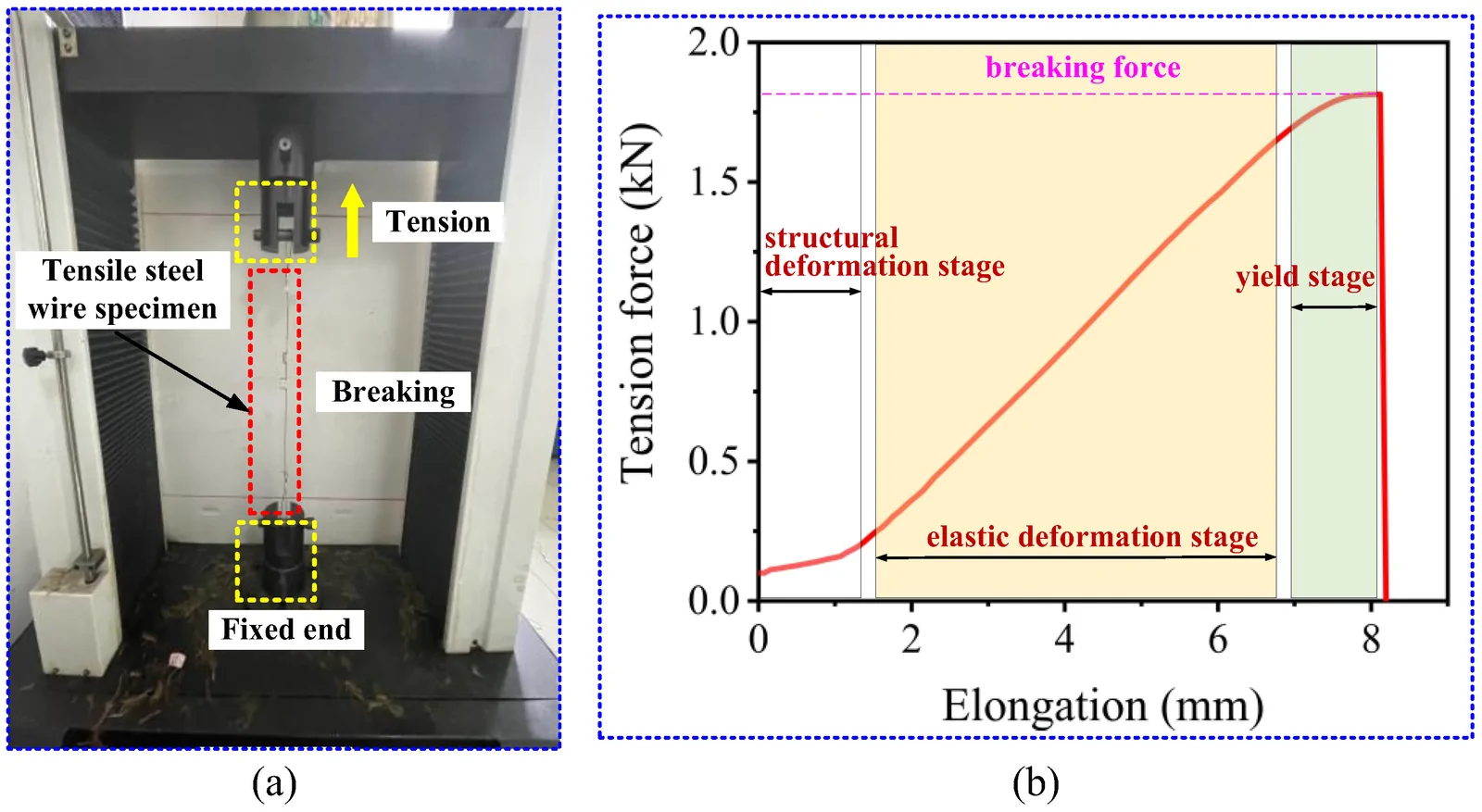
Testing methods for service performance: (**a**) universal tensile testing machine, (**b**) tensile breaking force-deformation curve.

After fretting friction and wear tests under different loads [7,35], fracture tests were conducted using a universal tensile testing machine (Zhongchuang Industry, Jinan, Shandong, China). to obtain the mechanical property degradation curves of the tensile strength (Figure 3). Both ends of the worn wire were fixed to the upper and lower grips of the testing machine, respectively. The lower end was kept stationary, while the upper end was stretched slowly. The force and deformation data of the wire were recorded using a displacement sensor and a force sensor. The parameters of the tensile fracture tests are listed in Table 2. By analyzing the force-elongation curves of worn wires under different test conditions, the relationship between fretting wear parameters and mechanical property degradation of the wires can be obtained. It is worth noting that the friction and wear parameters between wires were all derived from the dynamic characteristic analysis results. Since both the relative sliding contact and contact force between wires vary dynamically, the friction and wear test parameters were selected within the dynamic response range. The transverse contact loads between wires were set as 80 N, 160 N, 240 N, and 320 N, respectively, based on the dynamic characteristic analysis results. The initial relative sliding displacement was set to ±400 μm, while the stable sliding displacement during the steady wear stage was approximately 20–50 μm. It should be noted that the initial displacement amplitude applied by the testing machine is not completely equivalent to the actual stable sliding displacement at the wire contact interface. Due to specimen clamping, structural elastic deformation, and contact hysteresis, the stable interfacial sliding amplitude is significantly lower than the externally imposed displacement, and eventually stabilizes within the range of 20–50 μm. This range is basically consistent with the relative sliding displacement between internal wires obtained from the dynamic calculation.

### 2.5. Reliability Evaluation Method

The rainflow counting method was used to count and statistically analyze the service working stress of the wire and the time history of a single hoisting cycle. The number of occurrences corresponding to each stress mean value was obtained, and the distribution form of the stress was determined.

Let *Q* denote the working stress of the in-service wire, and let *W*(*n*) denote the residual strength of the damaged wire at the *n*-th fretting cycle. The failure criterion of the wire is defined as Equation (7):

(7) W(n)≤Q

The definition of the performance function *g*(*n*) is Equation (8):

(8) g(n)=W(n)−Q

When *g*(*n*) > 0, the steel wire is in the normal service stage; when *g*(*n*) ≤ 0, the steel wire fails. The reliability of the steel wire *R*(*n*) is given by Equation (9):

(9) R(n)=P[W(n)>Q]

If both the working stress and the residual strength follow a normal distribution, then Equations (10) and (11) are satisfied:

(10) $$Q\sim N\left(\mu_{Q},{\sigma_{Q}}^{2}\right)$$

(11) $$W\left(n\right)\sim N\left(\mu_{w\left(n\right)},{\sigma_{W\left(n\right)}}^{2}\right)$$

where $\mu_{Q}$ is the mean value of the working stress, $\sigma_{Q}$ is the standard deviation of the working stress, $\mu_{w\left(n\right)}$ is the mean value of the strength, and $\sigma_{W\left(n\right)}$ is the standard deviation of the strength.

**Figure 4:**
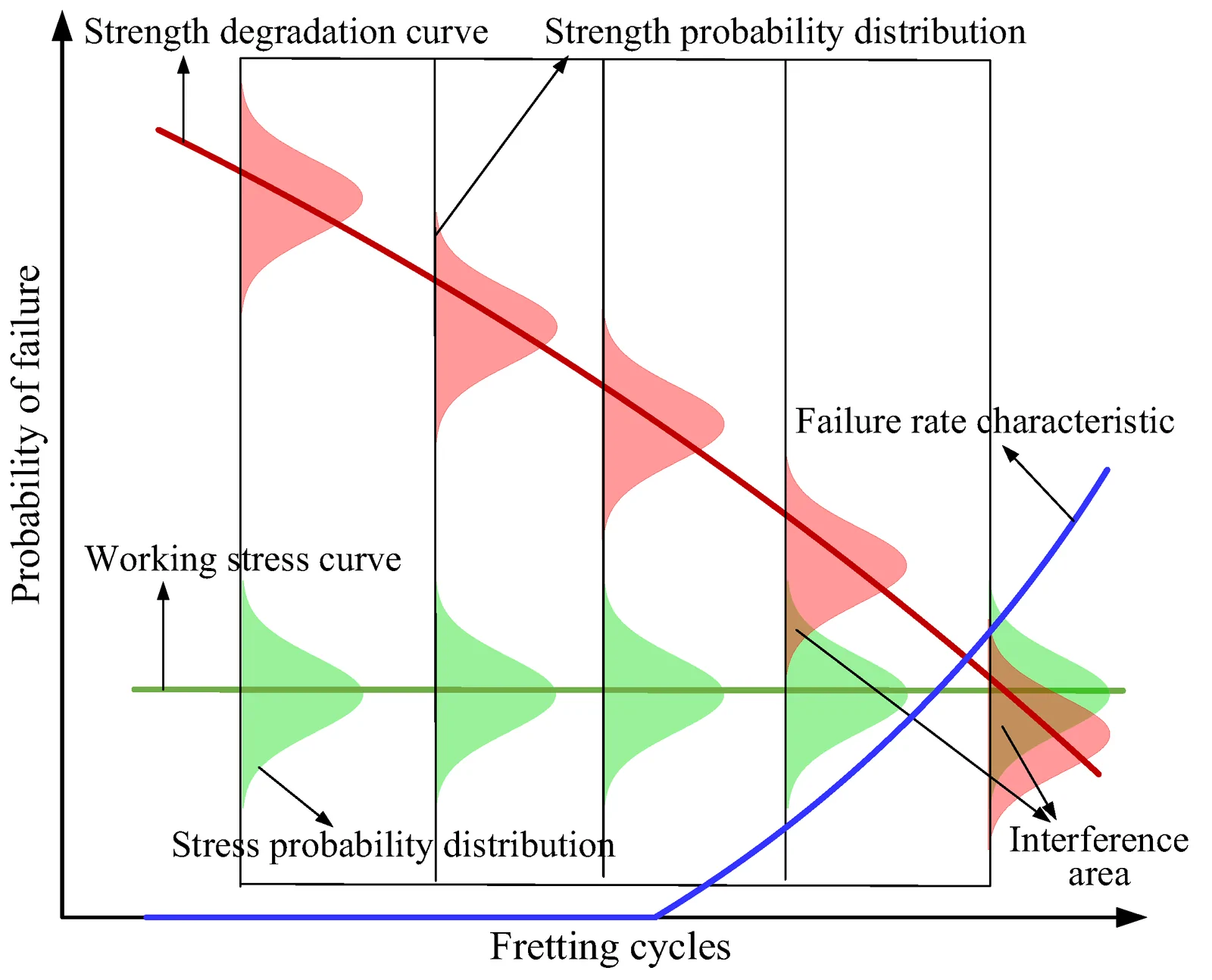
Dynamic evolution of stress-strength interference failure rate.

Using the distribution curves of wire stress and strength, combined with the stress-strength interference model shown in Figure 4, the reliability of the wire can be calculated. Furthermore, based on the strength degradation curve of the wire, the dynamic evolution of wire reliability throughout the entire service process can be obtained.

The reliability index *β*(*n*) can be expressed as Equation (12):

(12) $$\beta \left(n\right)=\frac{\mu W\left(n\right)-\mu_{Q}}{\sqrt{{\sigma_{W\left(n\right)}}^{2}}+{\sigma_{Q}}^{2}}$$

After determining the distributions of stress and strength, the reliability was solved using the Monte Carlo simulation method. In MATLAB (R2023b), *N* random samples conforming to the probability distributions of the corresponding parameters were generated. A sufficiently large sample size is generally required in this process to ensure the reliability of the results. Therefore, the number of random samples was set to 10^5^. The number of failed samples among all simulated samples was then counted, and the wire reliability R(n) was obtained based on the failure frequency, as expressed in Equation (13):

(13) $$R\left(n\right)=1-\frac{N_{f}}{N}$$

where *N_f_* is the number of failed samples, *N* is the total number of samples.

By repeatedly calculating the strength distributions at different fretting cycles on the strength degradation curve, the dynamic reliability curve of the damaged wire can be obtained.

## 3. Results and Discussion

### 3.1. Dynamic Response Characteristics

**Figure 5:**
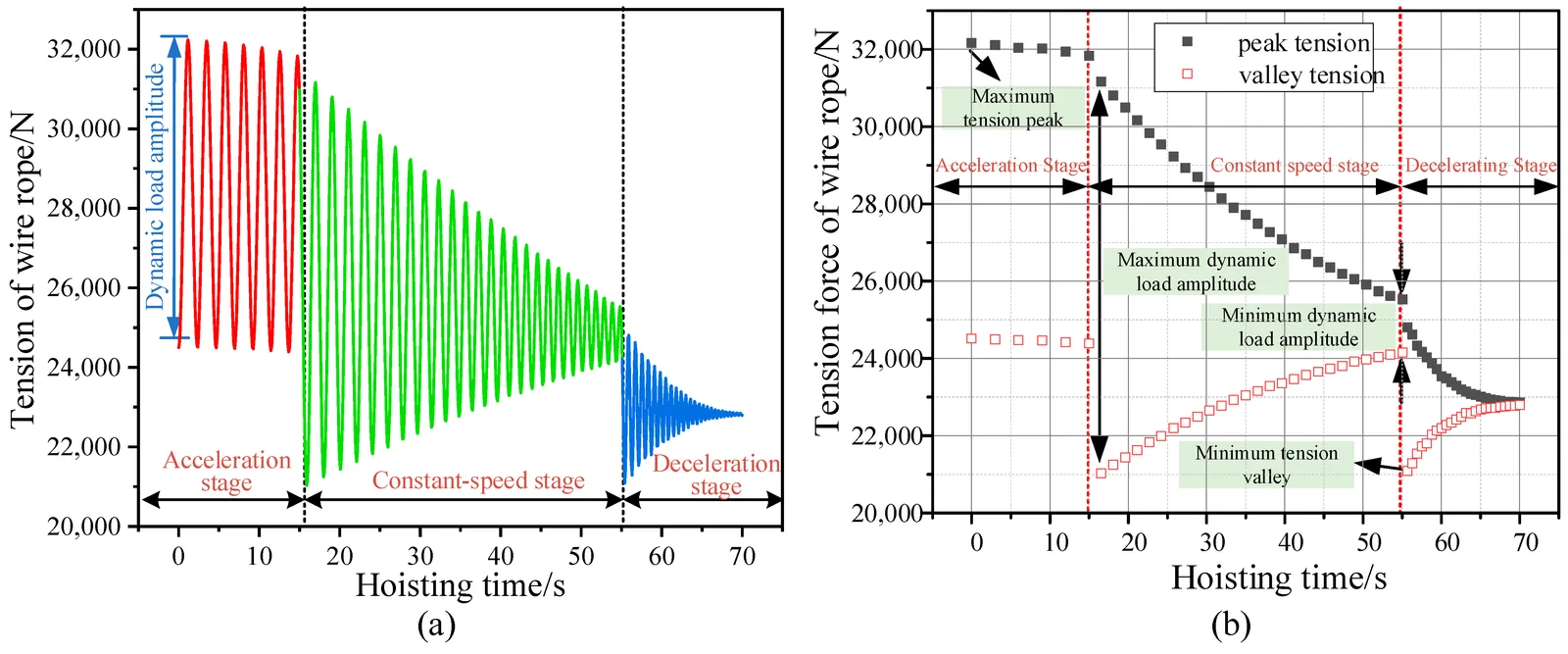
Tension variation in the wire rope during hoisting: (**a**) dynamic load variation curve, (**b**) amplitude variation in dynamic load.

Figure 5a shows the variation curve of wire rope tension during hoisting obtained using Simulink. It can be seen that the tension curve can be mainly divided into three parts, corresponding to the acceleration stage, constant-speed stage, and deceleration stage of the hoisting process, respectively. Sudden changes occur in the transition regions between adjacent stages, which are mainly caused by the abrupt change in acceleration during hoisting, resulting in inertial force variation and flexible impact of the wire rope. Overall, the tension curve exhibits a fluctuating trend, and the adjacent local maximum and minimum values of tension are used to characterize the dynamic load amplitude of the wire rope. During hoisting, the continuous variation in the total hoisted mass, caused by the decreasing suspended rope mass, together with the change in the equivalent stiffness of the wire rope, is the main reason for the continuous fluctuation of tension. In addition, it can be observed that the fluctuation frequency of the tension increases with hoisting time. This is because the rope length continuously decreases during hoisting, and the rope mass is correspondingly reduced, leading to an increase in the vibration frequency of the wire rope. Moreover, as the hoisting time increases, the rope length shortens and the equivalent mass decreases continuously, causing the overall tension curve to show a downward trend.

The tension fluctuation of the wire rope is one of the main causes of fretting damage between internal wires. The peak and valley points of the wire rope tension fluctuation curve were extracted to obtain the dynamic load amplitude. Figure 5b shows the variation process of the tension extrema and dynamic load amplitude of the wire rope during the complete hoisting process. The results show that the tension peaks decrease continuously with increasing hoisting time. This is because the reduction in suspended rope length decreases the equivalent mass of the wire rope, thereby reducing the peak rope tension. During the acceleration stage, relatively large tension fluctuations occur. This is attributed to the flexible impact force induced by the sudden acceleration, which causes tension oscillation in the wire rope. Meanwhile, the suspended rope length is relatively long at this stage, resulting in a large tension fluctuation amplitude. The maximum dynamic load amplitude appears at the initial stage of the constant speed process. When the system enters the constant-speed stage, the acceleration changes abruptly, causing the wire rope to be subjected to a large inertial force and flexible impact force. As a result, the vibration of the wire rope is further intensified, and the dynamic load fluctuation increases. With increasing hoisting time, the dynamic load amplitude gradually decreases. This is because the effects of flexible impact and inertial force are gradually attenuated during the constant-speed stage, leading to a progressive reduction in rope tension fluctuation. After entering the deceleration stage, the abrupt change in acceleration causes the wire rope to experience inertial force and enter an instantaneous “slack” state, which reduces the overall tension level while increasing the tension fluctuation amplitude. During the deceleration stage, as the hoisting velocity gradually approaches zero, the tension fluctuation of the wire rope also decreases continuously.

**Figure 6:**
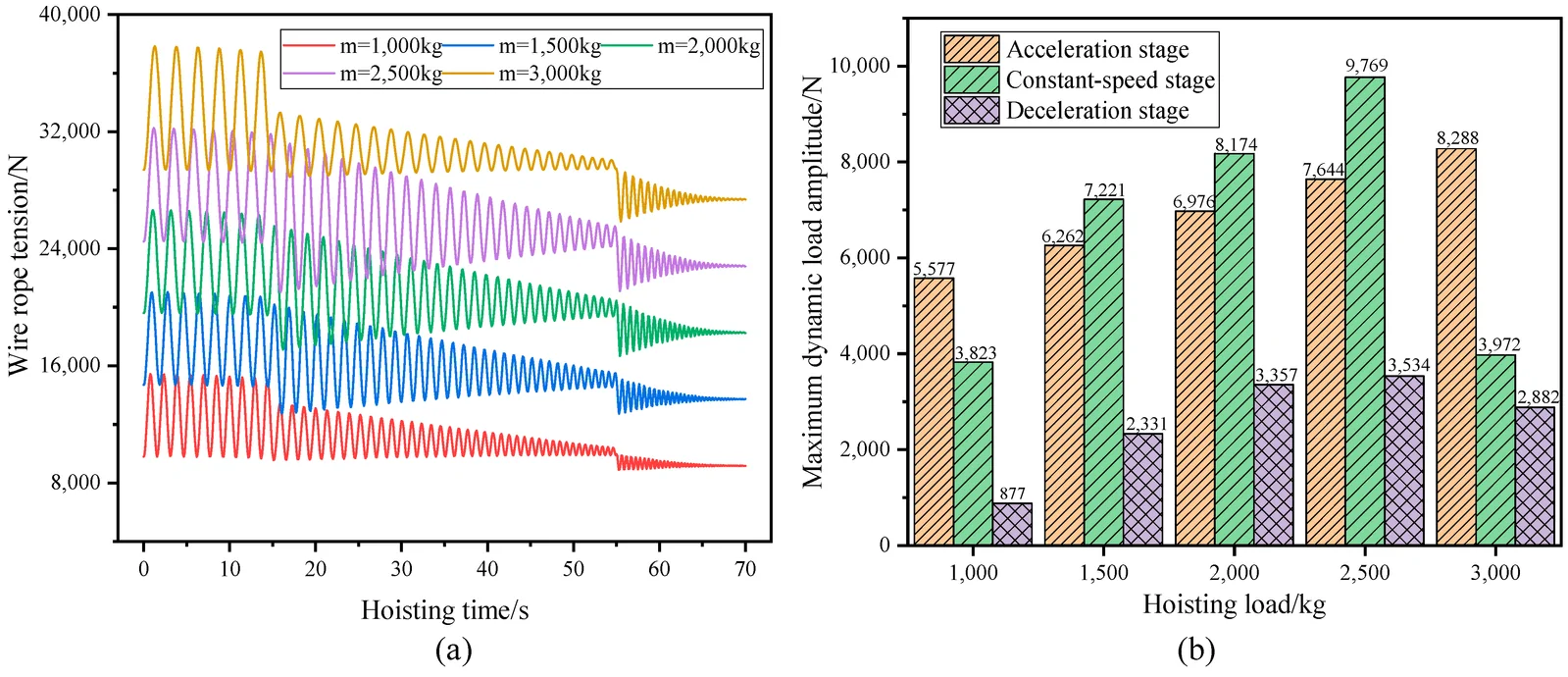
Tensile properties of wire rope under different hoisting loads: (**a**) tension variation curve, (**b**) maximum dynamic load amplitude.

Figure 6a shows the variation curves of wire rope tension under different hoisting loads during the hoisting process. The hoisting load ranges from 1000 kg to 3000 kg. It can be observed that the tension curves of the wire rope under different hoisting loads exhibit generally similar variation trends, and sudden changes occur at the transition stages among acceleration, constant speed, and deceleration. With increasing hoisting load, the overall wire rope tension shows an increasing trend. In addition, since the vibration frequency (*ω*_2_ = *E_R_A_S_*/[*L*(*t*)(*m_R_* + *ρL*(*t*)/3)]) is inversely related to the hoisting mass and rope length, the fluctuation frequency of wire rope tension decreases as the hoisting load increases, whereas the vibration frequency of the wire rope increases with increasing hoisting time. Figure 6b shows the maximum dynamic load amplitudes of the wire rope under different hoisting loads. The maximum dynamic load amplitude first increases and then decreases slightly with increasing hoisting load. When the hoisting load increases from 1000 kg to 2500 kg, the maximum dynamic load amplitude increases from 5577 N to 9769 N, and then decreases to 8288 N when the hoisting load reaches 3000 kg. This variation trend is consistent with that observed in the constant-speed stage and is related to the variation in wire rope stiffness and the magnitude of flexible impact. From the perspective of the three hoisting stages, the maximum dynamic load amplitude of the wire rope generally occurs in the constant speed and acceleration stages, whereas the dynamic load amplitude in the deceleration stage is usually relatively small. This is mainly because the suspended rope length is longer during the acceleration and constant-speed stages, and the total hoisting mass, including the terminal load and 1/3 of the rope weight, is larger. Consequently, greater inertial forces and flexible impact forces are generated, resulting in larger tension fluctuations in the wire rope.

### 3.2. Internal Steel Wire Contact Mechanical Properties

**Figure 7:**
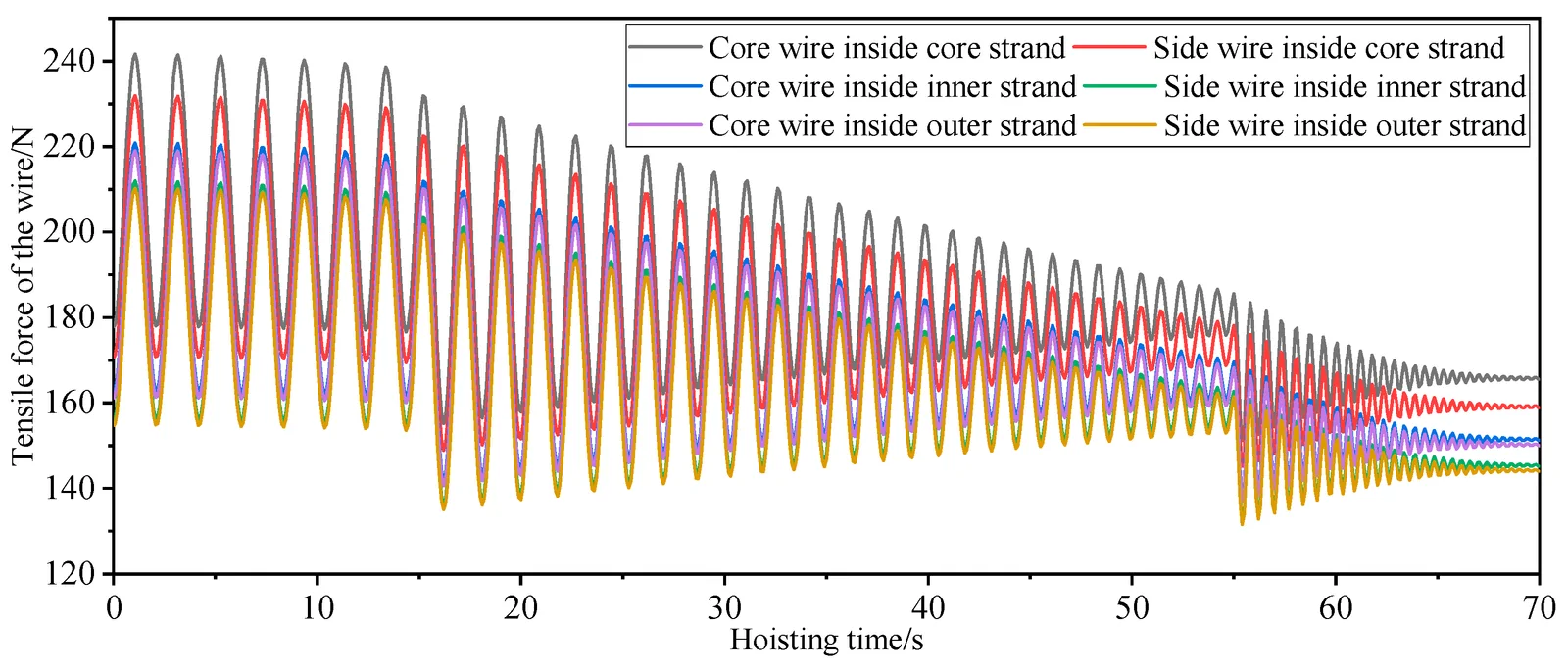
Variation in internal steel wire tension with hoisting time under different hoisting loads.

Figure 7 shows the variation in tensile force of wires at different positions inside the hoisting wire rope with hoisting time. It can be observed that the wires at different positions exhibit similar tensile force variation trends, which are consistent with the variation trend of the overall wire rope tension. According to the acceleration, constant speed, and deceleration stages, the tensile force curves can also be divided into three parts, with sudden changes occurring in the transition regions between adjacent stages. Due to the tension fluctuation of the wire rope, each wire correspondingly exhibits tensile force fluctuations, and the amplitude and frequency of these fluctuations are consistent with those of the wire rope tension. In addition, the tensile force levels of wires at different positions are different, following the order: core wire of the core strand > side wire of the core strand > core wire of the inner strand > side wire of the inner strand > core wire of the outer strand > side wire of the outer strand.

**Figure 8:**
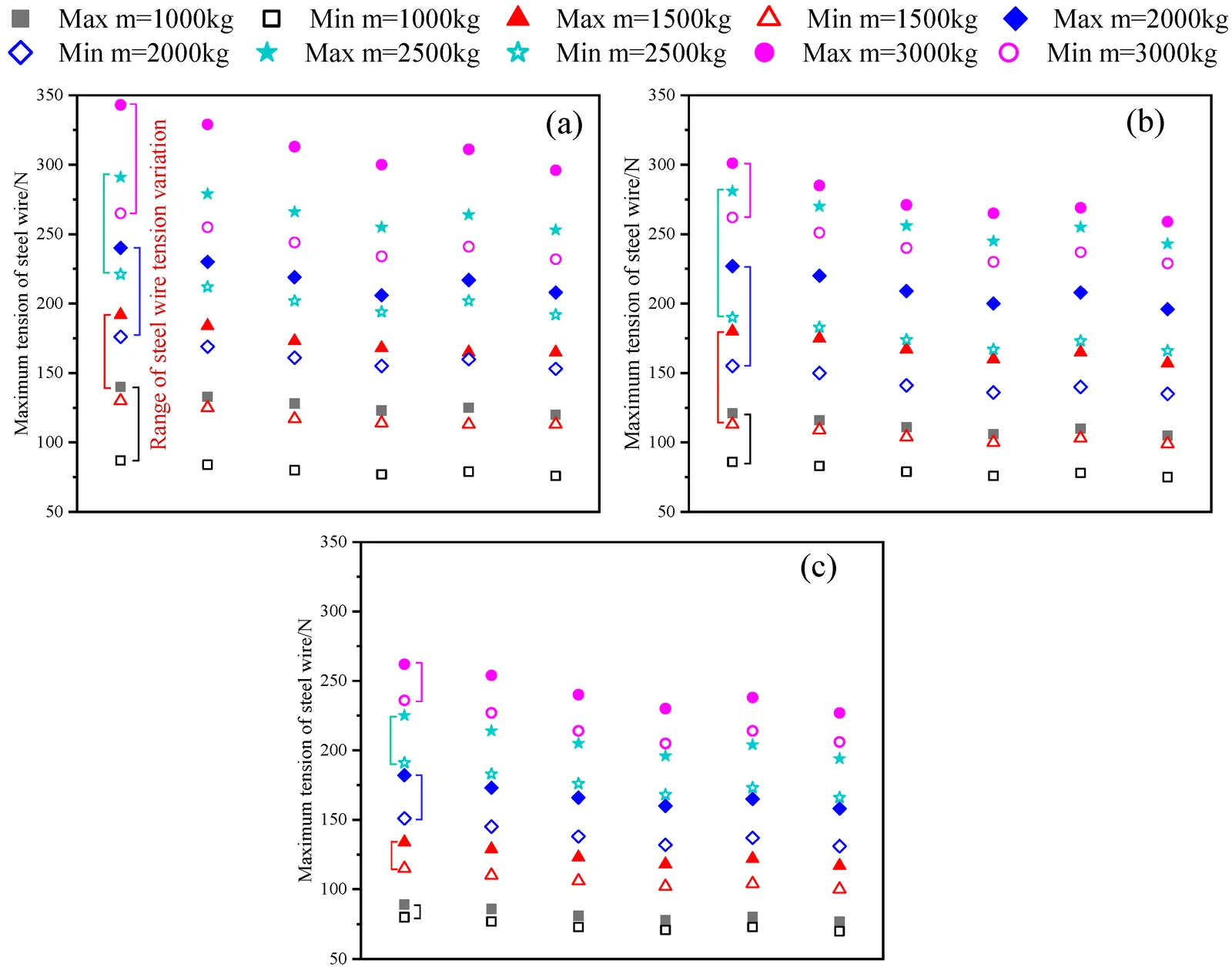
Maximum tension of steel wires at different positions inside the wire rope: (**a**) acceleration stage, (**b**) constant-speed stage, (**c**) deceleration stage.

Based on the tensile force variation curves of the wires, the maximum and minimum tensile forces of wires at different positions inside the wire rope under different hoisting loads and hoisting stages were analyzed, as shown in Figure 8. It can be observed that the extrema of wire tensile force are relatively large during the acceleration stage and relatively small during the deceleration stage. With increasing hoisting load, both the maximum and minimum tensile forces of the wires increase. In addition, within the same strand, the tensile force of the core wire is greater than that of the side wires. This is because the core wire has a larger diameter, and under the same deformation condition, the wire with a larger cross-sectional area bears a higher tensile load. The difference between the maximum and minimum tensile forces of a wire is defined as the maximum dynamic load of the wire. It can be found that the dynamic load amplitudes of the wires are relatively large during the acceleration and constant-speed stages, ranging from 30 N to 82 N. In the deceleration stage, due to the lower hoisting velocity and shorter rope length, the tensile force fluctuation of the wires is smaller, and the dynamic load amplitude ranges approximately from 9 N to 34 N.

**Figure 9:**
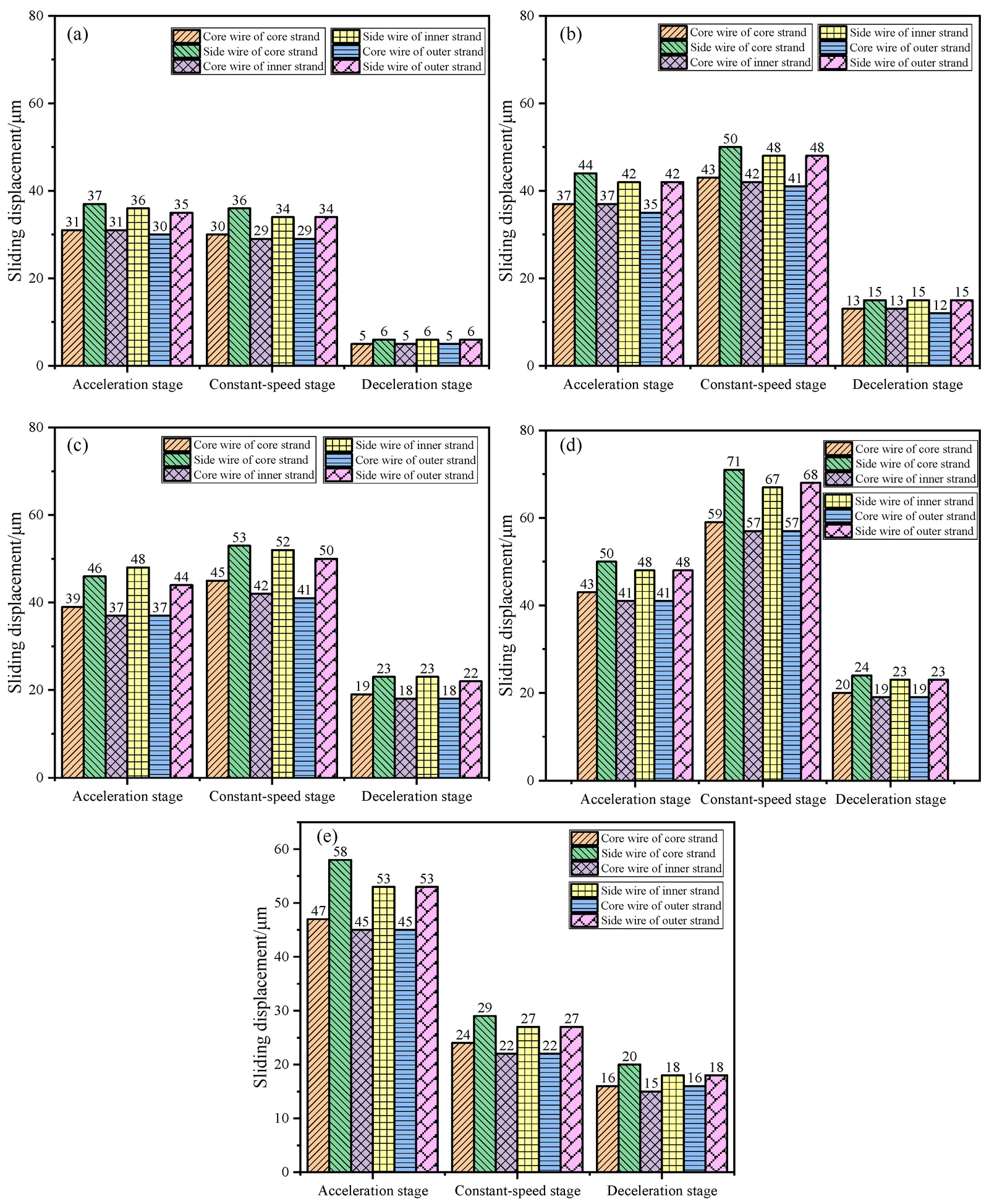
Slip amounts of individual wires within the wire rope under different hoisting loads and hoisting stages: (**a**) m = 1000 kg, (**b**) m = 1500 kg, (**c**) m = 2000 kg, (**d**) m = 2500 kg, (**e**) m = 3000 kg.

Figure 9 shows the evolution of sliding displacement of wires at different positions inside the wire rope under different hoisting stages and hoisting loads. Under different hoisting loads, the evolution trends of the sliding displacement between internal wires of the wire rope are basically consistent. Taking the results under a hoisting load of (m = 2000) kg as an example, the sliding displacement evolution at different hoisting stages and wire positions was analyzed. The sliding displacement of the wires is approximately 37–48 μm in the acceleration stage, 41–53 μm in the constant-speed stage, and 18–23 μm in the deceleration stage. Obviously, the sliding displacement between wires is relatively large in the acceleration and constant-speed stages, whereas it is relatively small in the deceleration stage. In addition, during the acceleration stage, the sliding displacements of the core wires in the core strand, inner strand, and outer strand are 39 μm, 37 μm, and 37 μm, respectively, while those of the side wires in the core strand, inner strand, and outer strand are 46 μm, 48 μm, and 44 μm, respectively. It can be found that, within the same strand, the sliding displacement of the core wire is slightly smaller than that of the side wires. The same tendency is also observed in the constant-speed and deceleration stages.

When the hoisting load increases from 1000 kg to 3000 kg, the sliding displacement ranges of the wires in the core strand, inner strand, and outer strand during the acceleration stage are 31–58 μm, 31–53 μm, and 30–53 μm, respectively. During the constant-speed stage, the sliding displacement ranges of the wires in the core strand, inner strand, and outer strand are 24–71 μm, 22–67 μm, and 22–68 μm, respectively. During the deceleration stage, the corresponding sliding displacement ranges are 5–24 μm, 5–27 μm, and 5–23 μm, respectively. It can be observed that, in the acceleration and deceleration stages, the sliding displacement between wires increases with increasing hoisting load. In the constant-speed stage, however, when the hoisting load reaches 3000 kg, the overall sliding displacement between wires decreases, which is attributed to the reduction in the dynamic load amplitude of the wire rope.

**Figure 10:**
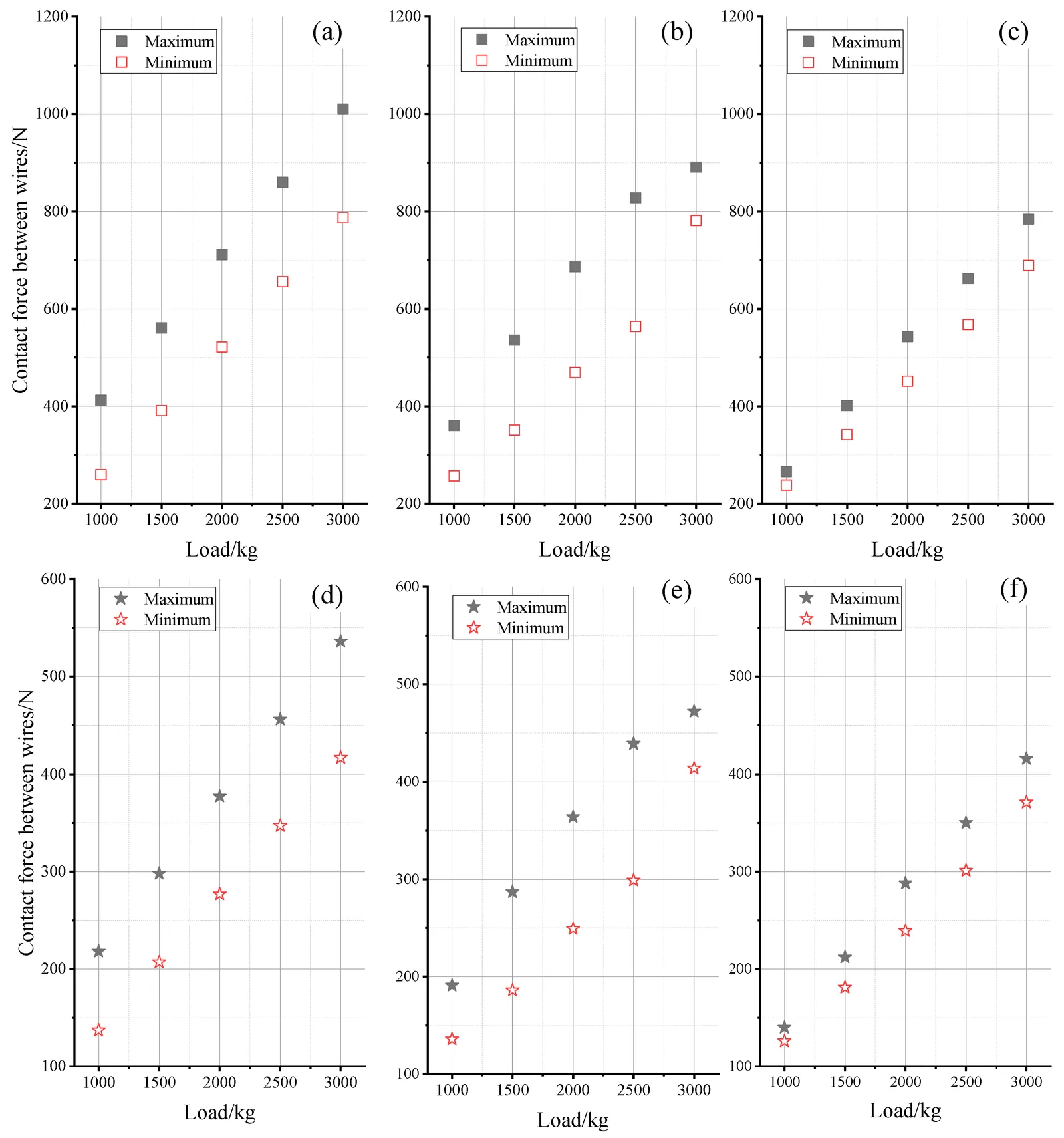
Contact force between wires in different strand layers: (**a**) core strand and inner strand–acceleration stage, (**b**) core strand and inner strand–constant-speed stage, (**c**) core strand and inner strand–deceleration stage, (**d**) inner strand and outer strand–acceleration stage, (**e**) inner strand and outer strand–constant-speed stage, (**f**) inner strand and outer strand–deceleration stage.

Figure 10 shows the evolution of the contact force between wires in different strand layers, namely between the core strand and inner strand and between the inner strand and outer strand, under different hoisting loads and hoisting stages. Obviously, the evolution trends of the inter-wire contact force under different hoisting loads are generally similar. When the hoisting load is 1000 kg, the contact force between the wires of the core strand and inner strand ranges from 260 N to 412 N, 257 N to 360 N, and 238 N to 266 N in the acceleration, constant-speed, and deceleration stages, respectively. The contact force between the wires of the inner strand and outer strand ranges from 137 N to 218 N, 136 N to 191 N, and 126 N to 140 N in the corresponding stages, respectively. It can be found that the contact force between the wires of the core strand and inner strand is significantly higher than that between the wires of the inner strand and outer strand, and is also higher than that between wires at other positions inside the wire rope. This is because the contact form between the wires of the core strand and inner strand is mainly point contact. In addition, after the outer and inner strands are tensioned, the squeezing force toward the rope center is superimposed onto the wires of the core strand.

Furthermore, the contact force level and variation range between wires in different strand layers are larger in the acceleration stage than in the constant-speed stage, and are the smallest in the deceleration stage. This is consistent with the contact force variation characteristics of wires at other positions. When the hoisting load increases to 3000 kg, the contact force ranges between the wires of the core strand and inner strand are 787–1010 N, 781–891 N, and 689–784 N in the acceleration, constant speed, and deceleration stages, respectively. The corresponding contact force ranges between the wires of the inner strand and outer strand increase to 417–536 N, 414–472 N, and 371–416 N, respectively. Clearly, the contact force between wires in different strand layers increases with increasing hoisting load. When the hoisting load ranges from 1000 kg to 2500 kg, the contact force range between wires in different strand layers increases with increasing load. However, when the hoisting load reaches 3000 kg, the contact force range between wires decreases during the constant-speed stage, which is related to the reduction in the rope tension fluctuation and the tensile force amplitude between wires.

### 3.3. Degradation of the Mechanical Properties of Damaged Steel Wires

**Figure 11:**
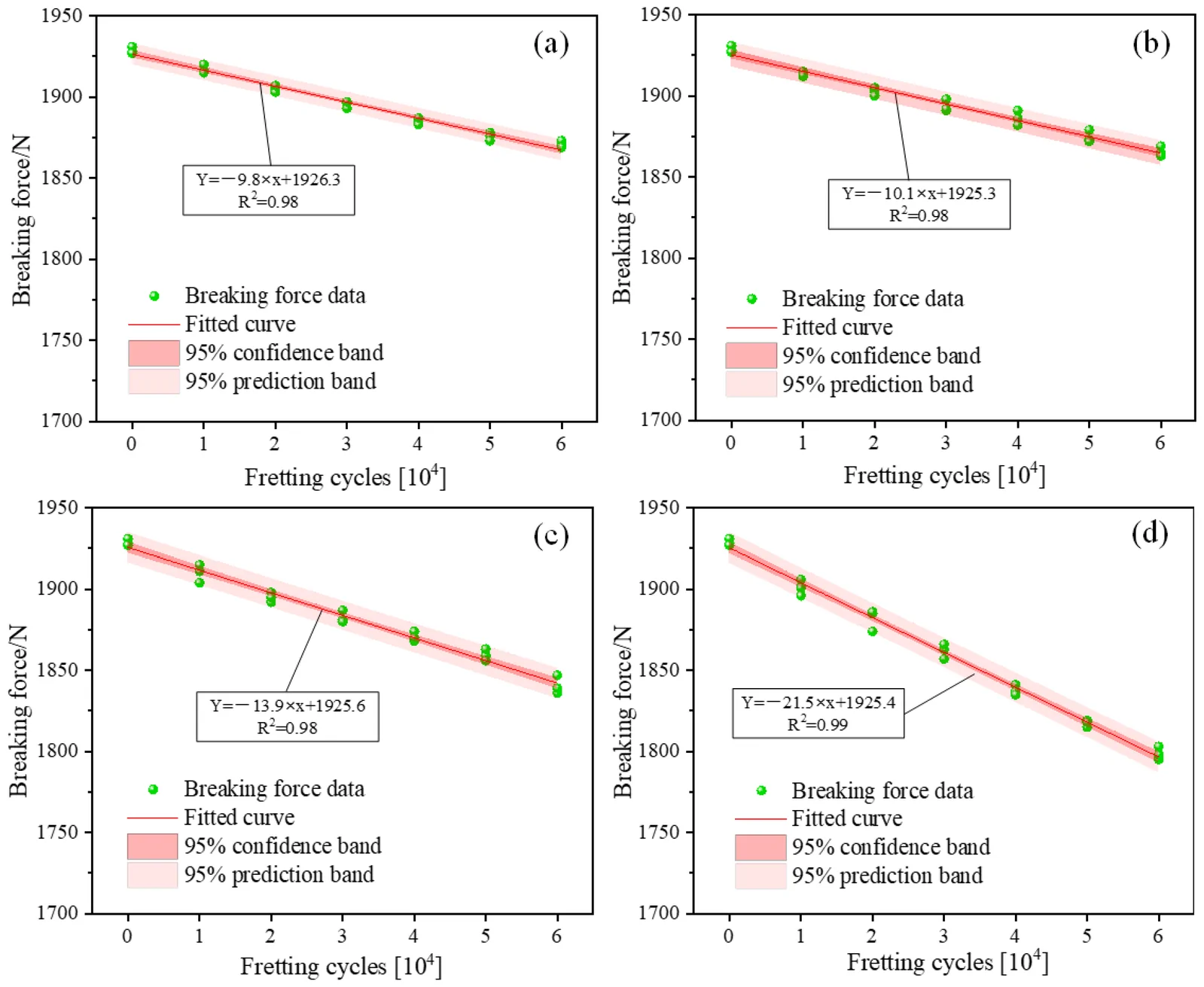
Degradation law of steel wire breaking force in service under different transverse load conditions: (**a**) 80 N, (**b**) 160 N, (**c**) 240 N, (**d**) 320 N.

Figure 11 shows the degradation curves of the breaking force of in-service wires under different transverse load conditions. It can be observed that the wire strength decreases continuously with increasing fretting cycles. When the transverse load increases from 80 N to 320 N, the degradation rate of wire strength with fretting cycles increases progressively. At a fretting cycles number of 60,000, the breaking forces of the wires are approximately 1871 N, 1863 N, 1841 N, and 1799 N, respectively. This is because the increase in transverse load enhances the contact force between wires, thereby increasing the wear rate between wires and further aggravating wire wear and strength degradation. By fitting the breaking force values of the wires under different fretting cycles, the mechanical property degradation curves of damaged wires under different transverse loads were obtained. It can be found that the mechanical property degradation curves of the wires approximately follow a linear decreasing trend, expressed as (Y = ax + b), with fitting correlation coefficients not less than 0.98. With increasing transverse load, the fitted value of “a” decreases continuously from −9.8 to −21.5, whereas the value of “b” changes only slightly. Under transverse loads of 80, 160, 240, and 320 N, the strength degradation curves are expressed as “Y = −9.8x + 1926.3”, “Y = −10.1x + 1925.3”, “Y = −13.9x + 1925.6”, and “Y = −21.5x + 1925.4”, respectively.

### 3.4. Evaluation of the Service Reliability of Damaged Steel Wires

**Figure 12:**
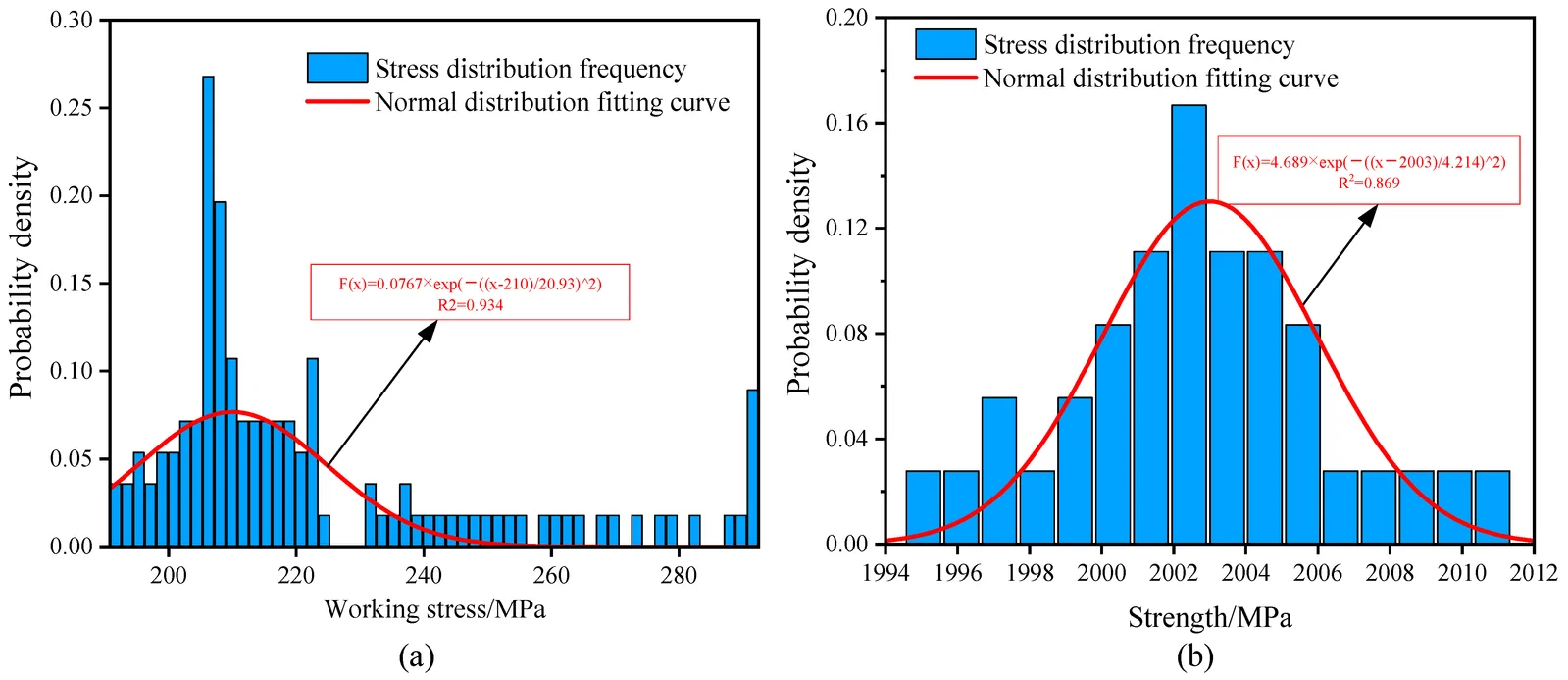
Stress distribution and strength distribution of service steel wire: (**a**) work stress, (**b**) degradation strength.

Figure 12a shows the stress distribution of the in-service wire obtained using the rainflow counting method. It can be seen that the stress distribution exhibits a certain regularity, with most stresses concentrated between 200 MPa and 240 MPa. A pronounced peak appears near 210 MPa, indicating that stresses in this region occur with the highest frequency, after which the probability density shows a decreasing trend. From the probability density distribution of the mean stress of the wire, it can be observed that the stress follows a normal distribution. The empirical stress distribution exhibits a pronounced frequency peak near 210 MPa and a dispersed upper-stress tail. Although the higher stress values occur less frequently, they increase the arithmetic mean of the distribution. The mean and standard deviation calculated from the stress data are 241.63 MPa and 26.61 MPa, respectively. Thus, the center of the histogram-fitted curve near 210 MPa should be distinguished from the arithmetic mean of the stress data. The reliability analysis adopts a normal approximation defined by the data-derived mean and standard deviation, rather than by the center parameter of the histogram-fitted curve. Similarly, the strength of the in-service wire at a specific fretting cycle was statistically analyzed, and its probability density distribution and cumulative distribution function were obtained. Figure 12b shows the strength distribution at 20,000 fretting cycles. It can be found that the strength also follows a normal distribution, with a mean value of 2002.7 MPa and a standard deviation of 3.1959 MPa. Based on the above distribution characteristics and method, the stress and strength distributions of wires under different transverse loads were further investigated. The mean working stress of the wire is 241.6 MPa, with a coefficient of variation of 0.1101. The strength distributions of the wires under different transverse loads all conform to normal distributions, and the coefficients of variation from 80 N to 320 N are 0.0015, 0.0016, 0.0021, and 0.0029, respectively.

**Figure 13:**
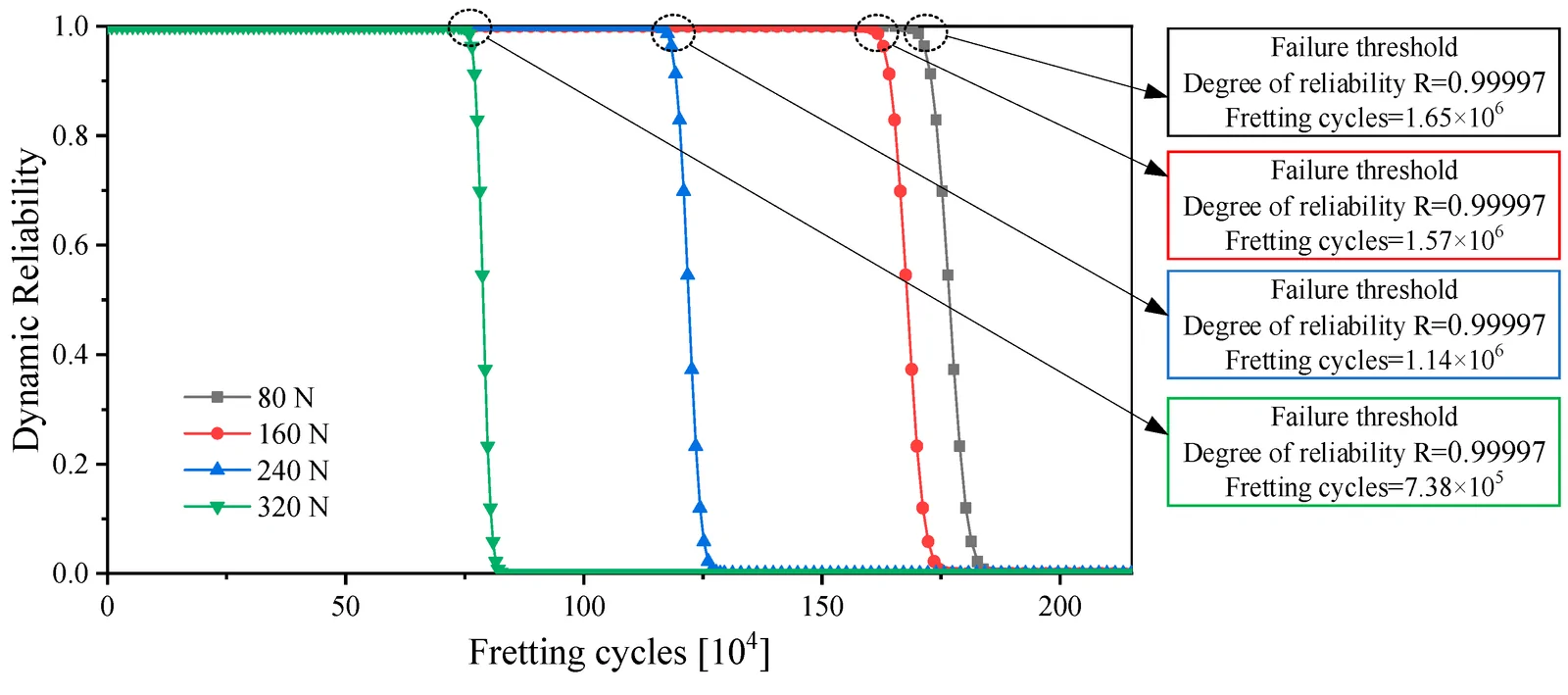
Dynamic reliability curves of steel wire in service under different transverse loads.

Figure 13 shows the dynamic reliability curves of in-service wires under different transverse loads of 80 N, 160 N, 240 N, and 320 N. It can be observed that, at the initial stage of fretting cycles, the dynamic reliability under all load conditions remains equal to 1, indicating that the wire has very high reliability at this stage and failure is almost unlikely to occur. With increasing fretting cycles, the reliability of the wire begins to decrease abruptly after reaching a certain critical failure point. This is because the accumulation of wear and fatigue damage in the wire increases with the number of fretting cycles, resulting in a decrease in reliability. In this study, the characteristic point before the rapid decrease in reliability is defined as the critical failure point, or (R = 0.99997) is adopted as the onset criterion for reliability degradation. Under the transverse load of 80 N, the critical failure point occurs at approximately 1.65 × 10^6^ cycles. Under transverse loads of 160 N and 240 N, the critical failure points occur at approximately 1.57 × 10^6^ and 1.14 × 10^6^ cycles, respectively, while, under the transverse load of 320 N, the critical failure point occurs at approximately 7.38 × 10^5^ cycles. These results indicate that a higher transverse load leads to fewer fretting cycles before wire failure, namely a shorter service life of the wire.

When the number of fretting cycles approaches the failure point, the dynamic reliability rapidly decreases to nearly 0, indicating that the reliability of the wire deteriorates sharply near the failure point and that failure eventually occurs. In addition, with increasing transverse load, the decline rate of the reliability curve also increases, which means that after the wire reaches the critical failure point, its reliability decreases more rapidly and complete failure, corresponding to zero reliability, is reached sooner. These findings demonstrate that a higher transverse load can significantly reduce the service cycles of the wire and thus greatly shorten its service life.

The linear degradation relationships are used here as an empirical approximation of the average loss of residual tensile strength per fretting cycle under each prescribed transverse load. This choice is supported by the approximately linear trends measured over the investigated cycle range and provides a consistent basis for comparing the effects of transverse load on reliability evolution. Extension of these relationships assumes that the average degradation rate remains unchanged under the same loading and contact conditions. The critical cycle counts in Figure 13 therefore represent conditional predictions of the stress-strength interference model under continued linear degradation. Their purpose is to characterize the predicted transition to rapid reliability decline and compare the investigated loading cases, rather than directly prescribe allowable service lives for an operating hoisting rope.

## 4. Conclusions

In this study, the internal wires of an 18 × 7 + IWS mine hoisting wire rope were taken as the research object. The time-varying dynamic characteristics of rope tension, as well as the residual strength degradation and dynamic reliability evolution of damaged wires under tension, were investigated. The main conclusions are as follows:

- During mine hoisting, the wire rope tension exhibits pronounced time-varying fluctuation characteristics. Large dynamic loads are generated at the acceleration-to-constant-speed and constant-speed-to-deceleration transition stages due to abrupt changes in acceleration. As the suspended rope length and equivalent mass decrease, the overall tension decreases, while the vibration frequency increases. Relatively large dynamic loads are mainly concentrated in the acceleration stage and the initial period of the constant-speed stage.
- The inter-wire contact load increases with increasing hoisting load, whereas the sliding displacement exhibits a stage-dependent, non-monotonic response to hoisting load. In particular, during the constant-speed stage, the sliding displacement decreases when the hoisting load increases to 3000 kg. After the overall rope tension is transmitted through the helical structure, significant positional differences occur in the internal wire forces and sliding behavior. The tensile force of the wires generally follows the order of core wire in the core strand being the largest and side wire in the outer strand being the smallest. The dynamic tensile force amplitude of the wires in the acceleration and constant-speed stages is approximately 30–82 N, which is significantly higher than that in the deceleration stage, which ranges from 9 to 34 N.
- Inter-wire sliding displacement is greater during the acceleration and constant-speed stages than during the deceleration stage. At a hoisting load of 2000 kg, the corresponding displacement ranges are 37–48 μm, 41–53 μm, and 18–23 μm, respectively. Increasing hoisting load increases the inter-wire contact force, but does not produce a monotonic increase in sliding displacement: at 3000 kg, the constant-speed-stage displacement decreases with the reduced dynamic load amplitude. The highest contact forces occur between the core strand and the inner strand, identifying this interface as a region of concentrated contact loading.
- The breaking force of damaged wires decreases approximately linearly with increasing fretting cycles. Under transverse loads of 80–320 N, the strength degradation slope increases from −9.8 to −21.5. The reliability analysis shows that the critical cycle at which reliability begins to decrease rapidly decreases from 1.65 × 10^6^ to 7.38 × 10^5^, indicating that an increase in transverse load significantly shortens the safe service period.

## Figures and Tables

**Table 1:** Lay parameters and structural parameters of wire rope.

Parameters	Unit	Values
Diameter of wire rope	[mm]	15.3
Diameter of rope strand	[mm]	3.1
Diameter of core wire	[mm]	1.1
Diameter of side wire	[mm]	1
Lay length of inner strand	[mm]	63.3
Lay length of outer strand	[mm]	121.3
Wire lay length	[mm]	32.6
Lay angle of inner strand	[°]	17.1
Lay angle of outer strand	[°]	17.8
Lay angle of wires	[°]	11.6
Number of strands	[-]	19
Lay method	[-]	regular lay
Number of core wires in a single strand	[-]	1
Number of side wires in single strand	[-]	6

**Table 2:** Mechanical properties testing machine parameters.

Parameters		Values
Specimen length	[mm]	400
Maximum stretching distance	[mm]	1000
Stretching velocity	[mm/min]	10
Pretightening force	[N]	100
Tensile accuracy	[N]	1
Maximum measurement range	[kN]	100
Test stop criteria	[-]	≤50%

## Data Availability

The original contributions presented in the study are included in the article; further inquiries can be directed to the corresponding author.
